# Relationship between distance to boron mine and exposure in cattle

**DOI:** 10.1007/s10653-025-02484-y

**Published:** 2025-04-15

**Authors:** Cagla Celebi, Huseyin Sen, Hasan Susar, Murat Celebi, Izzet Karahan

**Affiliations:** 1 Department of Pharmacology and Toxicology, Health Sciences Institute, Balıkesir University, Balıkesir, Turkey; 2 Department of Pharmacology and Toxicology, Faculty of Veterinary Medicine, Balıkesir University, Balıkesir, Turkey; 3 Department of Veterinary, Savaştepe Vocational School, Balıkesir University, Balıkesir, Turkey

**Keywords:** Boron, Residue, Exposure, ICP-OES, Mineral, Cattle

## Abstract

Boron, a valuable underground mineral whose usage area is increasing day by day, has been identified as an essential trace element in plant development. However, research on its usage for humans and animals is still ongoing. Recommended doses are physiologically involved in many systems, but excess boron can be risky for living organisms and the environment. Boron moves in an endless cycle between air, water, soil, and food, and can accumulate. Concentration can rise too much, especially in areas with boron mines. This situation causes contamination in the environment and directly affects human, animal and plant health. There is a paucity of research on the residue status of boron mineral, which is extensively mined and frequently used in certain regions, notably Turkey. In our study, we sought to ascertain the effects of proximity to boron mines on boron concentration in blood, urine, water, and feed in animals. In the study, 60 (20*3) cattle living in areas 0–5, 5–15 and 15–30 km away from the boron mine site were used. Blood and urine samples were taken from cattle. Boron concentrations were determined by sampling the feed and water they consumed. The results of the analyses demonstrated that boron levels in all samples were influenced by the distance to the mine. A statistically significant decrease was observed, particularly in drinking water and blood boron levels. This situation is a major risk factor, especially for living organisms in proximity to boron and other mines. It is also recommended to establish more comprehensive studies investigating the effects of boron concentration on living organisms.

## Introduction

Boron, which is found in nature in combination with different elements, especially oxygen, is the 51st most common element found in the earth's crust with an average concentration of 8 mg/kg (about 0.0008%). Although it is generally referred to as boron, there are nearly 250 varieties such as boric acid and borax. These compounds have crystalline or amorphous forms in different colors; concentrations of 5–150 mg/kg in soil, 0.5–9.6 mg/kg in seawater and 0.5–800 ng/m^3^ in air have been reported (Elevli et al., 2022; Ince et al., 2019). The main areas of use are industry and industrial activities. For this reason, the marketing of boron, known as a strategic mineral, is under control. Its main use is in the glass industry. In addition, it is also used as fertilizer and herbicide in the cleaning sector, ceramics, metallurgy and agricultural activities (Nielsen & Eckhert, 2020). The use of boron compounds is not limited to industry. Boron, which was used only as an antiseptic in medicine for a long time, has been playing many roles in mineral and hormone metabolism, development, defense system and wound-burn healing in recent years. In addition, the positive results of the effectiveness of boron nitride nanotubes against amyloid accumulation, the most important phenomenon in Alzheimer’s disease, have given hope to diseases that have no definitive treatment and to everyone working in this field (Aydın et al., 2022). Boron neutron capture therapy (BNCT), which has reported positive results in head and neck cancer, which is difficult to treat in humans, is being investigated as a new method in veterinary medicine with the increasing number of animals with cancer (Kanno et al., 2021; Kusaka et al., 2022).

Boron exposure is most commonly oral, but can also occur via dermal or inhalation routes. Regardless of the route of exposure, borates, which are converted to boric acid at physiological pH, are easily and almost completely absorbed and evenly distributed in body fluids by passive diffusion. Absorption of boron through healthy, intact skin is very low. Some studies have suggested that borates can also be absorbed through respiration and retained in the upper respiratory tract (Çoban-Karabağ et al., 2023; Kuru & Yarat, 2017). The excretion of borates ingested into the body occurs within 24 h, mostly through the kidney (largely by glomerular filtration). Some studies have reported that about 85% of boron is excreted in urine and that the amount of boron in urine may reflect most of the boron intake. Serum boron concentration is also thought to be mainly influenced by renal excretion (Kabu et al., 2015; Uluisik et al., 2018).

Boron, one of the seven essential trace elements in the natural world, is related to the survival and health of organisms. Boron, a trace mineral for humans and animals, is also required for various biological reactions in the body and is an essential element that acts as a cofactor for some enzymes as SOD, CAT, GSH (Türkez et al., 2007). Evidence suggests that dietary boron supplementation in physiological amounts in animal and human diets can have significant effects on various metabolic and physiological systems of the organism. It has been reported to help plant cell membranes maintain their structural and functional integrity and sustain normal reproductive development and substance transport. Little boron purchase intake is beneficial for the growth and development of animals and humans, as well as for disease prevention (Ince et al., 2017; Nielsen, 2014).

Metals are naturally present in the earth’s crust, where their composition varies with location, resulting in different concentrations. However, industrial processes, mining and agricultural activities can significantly increase metal concentrations (Rilwan et al., 2025; Waida & Rilwan, 2024). Metals with a density of more than 5 g/cm^3^ are called heavy metals and can have a health impact on living organisms and the environment. Moreover, if not degraded, they accumulate in the ecosystem and pose long-term risks to living organisms. Although some heavy metals are essential in trace amounts for biological processes, overexposure can lead to significant environmental and health problems. Pollution of the environment by these substances is a major problem, and their toxicity is also a major problem, compounding ecological, evolutionary, nutritional and environmental causes (Waida et al., 2022; Rilwan et al., 2020). Boron, a micronutrient and heavy metal, can also be toxic depending on the dose, duration and route of exposure. Case reports of high-dose poisonings have failed to identify a mechanism of toxicity for neurological and renal effects. Reproductive and developmental effects have been reported as the most sensitive toxic points. Boric acid and sodium borates are classified as toxic to reproduction and development under category 1B with the hazard statement H360FD (May cause harm to fertility. May cause harm to the unborn child) in the EU-CLP regulation. This situation has triggered environmental and occupational boron exposure studies on boron in countries with high boron exposure such as Turkey. The boundary between deficiency and toxicity of boron, which has a high coefficient of effect, is very thin (Duydu et al., 2023; Wang et al., 2018). For this reason, it is of great importance to reveal the toxic effects of these compounds, which are reported to have positive effects when taken into the body in recommended amounts. Toxic effects can often occur as a result of acute or, more rarely, chronic exposure. Boron concentration is a significant parameter in the context of chronic exposure. The consumption of foodstuffs cultivated in the specified area, the contamination of drinking water, the absorption of boron from the atmosphere, and, though to a lesser extent, from the skin, have the potential to pose a risk to living organisms in the region. (Duydu et al., 2018; Hadrup et al., 2021). For this reason, it is thought that research on boron should be increased in boron mining areas.

In response to the threat of boron to human health, the World Health Organization (WHO) has set a recommended boron intake value for humans (1–13 mg/kg). A total of 40% of boron intake by humans comes from drinking water. Boron can enter the aquatic environment in many ways such as mining, coal combustion, detergents, industrial pollutants, fertilizer/pesticide applications, etc. In 2011, WHO updated the limit value for boron concentration in drinking water to 2.4 mg/L. Different countries and regions around the world have established their own standards based on WHO recommendations according to their own contexts. In Turkey, which holds 73.5% of the world’s boron reserves (‘Regulation on Water Intended for Human Consumption’), the upper limit for boron is set at 1 mg/L for drinking water (Kuru et al., 2019; WHO, 2017). Recently, the incidence of boron in water resources has been reported to increase due to the growing global demand for boron in industrial plants. Water quality criteria have therefore become more stringent. Consequently, the determination of boron pollution in aquatic environments, remediation of boron contaminated waters and research in this field have attracted great interest in the last few years. The next most important source of boron intake, after water, is food. This element, which enters the metabolism of living organisms through nutrition and plays a direct or indirect role in many physiological events, can exhibit toxic effects in excess. According to the literature, the richest foods in terms of boron content are legumes. This situation can directly affect human health and also carries a risk for animals (Khaliq et al., 2018; Liu et al., 2022; Najid et al., 2021).

Previous studies have generally focused on humans. However, animals, especially those whose meat and dairy products are consumed, are an important link in the food chain and are directly related to public health. Considering the effects of boron exposure on living health, it is also important to accurately determine the boron concentration in the environment. In Turkey, which has high boron concentrations, there is insufficient data on the boron content of drinking water and feeds offered to animals. Therefore, in future studies, determination of boron levels in drinking water and foodstuffs consumed, evaluation of boron intake at different levels and determination of boron levels in different living fluids will provide useful information for the protection and improvement of health. In the light of the information presented above, the aim of this study was to determine the effects of distance to boron mines on boron concentrations in blood, urine, water and feed in animals. For this purpose, we focused on the exposure to boron, which is known to be abundant in Turkey, to animals in the region. The reported exposure in these animals and their products, especially those used for human consumption, is of direct public health concern.

## Material and methods

### Study groups and sample collection

The study was conducted in Bigadiç district of Balıkesir, located in the Marmara Region of Turkey. The region is one of the places where boron mines are found. The mine is located in Iskele town of the district and the study regions were selected from 0 to 5 km (short distance, SD), 5–15 km (medium distance, MD), 15–30 km (long distance, LD) locations. A total of 60 (20*3) cattle were studied, 20 cattle for each region. The owners of the animals included in the study were informed and written informed consents were obtained from them. This study was approved by Balıkesir University Local Ethics Committee (approval number: BAU-HADYEK 2022/8-3). The aim of this study is to determine the effects of distance to boron mines on boron concentrations in blood, urine, water and feed in animals.

Blood samples were collected from the tail vein of the cattle into anticoagulant-free tubes. Blood samples were centrifuged at 3500 g for 15 min and serum was removed. Sera collected in Eppendorfs were stored at − 80 °C until analyzed. Urine samples were collected from the animals by massage. Samples collected in sterile sample containers were stored at + 4 °C. Feed samples were collected in sterile sample bags of approximately 500 g. It was ensured that the feed samples were products grown in the region and/or silages prepared from these products. Water samples were collected from wells, drinkers and washing areas open to animal consumption. Water was collected in sterile and boron-free polyethylene sample containers, 50 ml + 50 ml in duplicates.

### Boron analysis

The method of Tokay and Bağdat (2022a) was modified for the analysis of the samples. After the samples were thawed, acid digestion method was applied to the liquidized sera. The method used 65% nitric acid and 35% hydrogen peroxide. The samples were heated for a certain period of time without boiling and the final volume was topped up to 10 ml. The same procedures were applied to urine and water samples. However, they were first filtered to remove impurities. Feed samples were collected as solids. They were then stored in aluminum containers and dried at 60 C and 80% air presentation for 48 h. The completely dried samples were ground and 200 mg of each weighed. HNO3 and H2O2 were added and the analysis time was waited for the determination of boron concentrations. The program power was reported as 90%. This process was performed on Berghof brand Speedwave Expert model device. The samples transferred to liquid form were subjected to boron analysis in the same device as the other samples. All prepared liquid samples were analyzed with Perkin-Elmer OPTIMA 7300 model ICP-OES device. The instrument specifications were determined according to Tokay and Bağdat (2022b).

### Operating conditions for ICP OES

A table of operating conditions for ICP OES has been created. This table is marked in red in the materials and methods section of the paper.

Determination of the analytes was carried out using Perkin Elmer 7300 DV model ICP OES (Waltham, MA, USA). The operating parameters for the spectrometer were set as recommended by the manufacturer, and the operating conditions were given in Table 1.

**Table 1:** Operating conditions for ICP OES

Torch viewing	Axial	Recalibration system	Hg lamb
RF power	1300 Watts	Nebulizer	Cross flow
Spray Chamber	Glass cyclonic spray chamber	Plasma gas flow	15 L min^−1^
Auxiliary gas flow	0.2 L min^−1^	Nebulization gas flow	0.8 L min^−1^
Sample flow rate	1.5 mL min^−1^	Delay time	60 s
Wash rate	1.5 mL min^−1^	Wash time	30 s

### Linearity

**Fig. 1:**
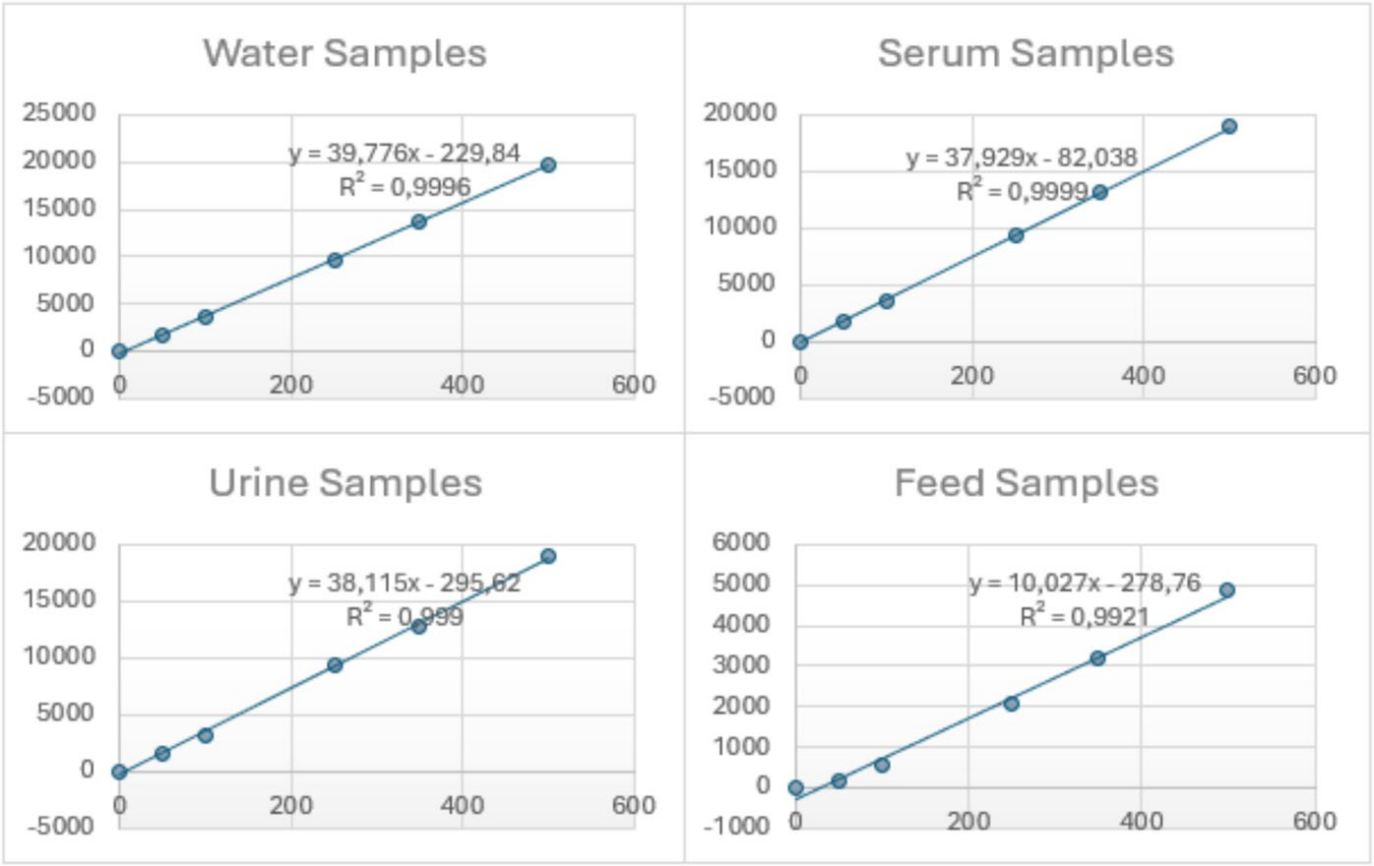
Calibration curves

For linearity, the correlation coefficient of the calibration curves at 50, 100, 250, 350, 500 ppb was found. The correlation coefficient was found to be (R2 ≥ 0.99). The calibration curves are given in Fig. 1.

### Recycling

The manually calculated recovery results are presented in Table 2.

**Table 2:** Recovery results of the analysis

Water samples			Serum samples			Urine samples			Feed samples		
Analyte (ppb)	Conc		Analyte (ppb)	Conc		Analyte (ppb)	Conc		Analyte (ppb)	Conc	
50	50	%100	50	50	%100	50	50	%100	50	50	%100
100	100	%100	100	100	%100	100	100	%100	100	100	%100
250	249.7	%99.72	250	250	%100	250	250	%100	250	250	%100
350	350	%100	350	353.1	%99.11	350	349.2	%99.77	350	350	%100
500	500	%100	500	500	%100	500	500	%100	500	500	%100
%99.94			%99.82			%99.95			%100		

### Limit of determination (LOD) and limit of measurement (LOQ)

In boron analysis, LOD values were 12.5669 µg/L for water, 5.3050 µg/L for serum, 20.6567 µg/L for urine and 57.8052 µg/L for feed. In boron analysis, LOQ values were determined as 41.8897 µg/L for water, 17.6834 µg/L for serum, 68.8556 µg/L for urine and 192.6843 µg/L for feed.

### Certipur ICP multi-element standard solution IV (CRM)

CRM for water samples is 250 ppb. 3 replicates were performed. Repeat results are 250, 250.9 and 249.7 ppb. The average CRM was calculated as 250.2 ppb.

CRM for serum samples is 350 ppb. 2 replicates were performed. Repeat results are 350 and 346.9 ppb. The average CRM was calculated as 348.45 ppb.

CRM for urine samples is 350 ppb. 2 replicates were performed. The repeat results are 350 and 335.7 ppb. The average CRM was calculated as 342.85 ppb.

CRM for feed samples is 500 ppb. 3 replicates were performed. The repeat results are all 500 ppb. The average CRM was calculated as 500 ppb.

### Statistical analysis

In this study, statistical analyses were conducted to compare boron concentrations in serum, urine, drinking water, and feed samples collected from different distances (0–5 km, 5–15 km, and 15–30 km). The assumption of normal distribution was evaluated by examining skewness and kurtosis values. According to Tabachnick and Fidell (2013), skewness and kurtosis values within the range of ± 1.5 indicate that the data meet the assumption of normal distribution. However, the data presented in the tables did not meet this assumption. Therefore, non-parametric tests were used for the analysis.

The Kruskal–Wallis H test was applied to evaluate differences between groups. When significant differences were detected, post-hoc analyses were performed to identify specific group differences. All statistical analyses were conducted using SPSS 30.0, and a significance level of *p* < 0.05 was considered.

## Results

Table 3 shows boron concentrations from various sources, including serum, urine, drinking water, and feed. In serum, boron concentration is on average 0.45 µg/L ± 0.27, ranging between 0.07 µg/L and 1.27 µg/L, with a positively skewed (1.492) and kurtotic (2.485) distribution. Urine shows significantly higher boron concentrations (mean: 11316.25 µg/L ± 15361.68), ranging from 1440 µg/L to 55,600 µg/L, indicating that urine might represent an important pathway for boron excretion (skewness: 2.200; kurtosis: 3.430). Boron in drinking water averages 887.49 µg/L ± 1551.84, varying between 47 µg/L and 6049 µg/L (skewness: 2.143; kurtosis: 3.612). In feed samples, boron concentration averages 534.85 µg/L ± 907.47, with a wide variation ranging from − 93.20 µg/L to 4620 µg/L, showing positive skewness (2.949) and a high kurtosis (9.223). Tabachnick and Fidell (2013) stated that skewness and kurtosis values within the range of ± 1.5 indicate that the data meet the assumption of normal distribution. When the table is examined, it has been determined that the data do not meet the assumption of normal distribution. The boron concentrations in the serum, urine, drinking water samples, and feed samples from Bigadic are compiled in Table 4.

**Table 3:** Boron concentration in serum, urine, drinking water and feed

	$\overline{{\varvec{x}} }$±SD	Min–Max	Skewness	Kurtosis
Serum (µg/L)	0.45 ± 0.27	0.07–1.27	1492	2485
Urine (µg/L)	11316.25 ± 15361.68	1440.00–55600.00	2200	3430
Drinking water (µg/L)	887.49 ± 1551.84	47.20–6049.00	2143	3612
Feed (µg/L)	534.85 ± 907.47	(− 93.20)–4620.00	2949	9223

$\overline{{\varvec{x}} }$±SD = mean ± standard deviation

**Table 4:** Comparison of data by distance

	0–5 km	5–15 km	15–30 km		
	Median (Min–Max)	Median (Min–Max)	Median (Min–Max)	H	*p*
Serum (µg/L)	0.54 (0.23–1.27)^a^	0.37 (0.07–0.49)^b^	0.45 (0.16–0.84)^ab^	9.929	**0.007**
Urine (µg/L)	10452.5 (1440.00–55600)	3319.5 (2014–8229)	4929 (2507–13765)	5.373	0.068
Drinking water (µg/L)	1285 (47.2–6049)^a^	93.20 (66.1–1466)^ab^	73.75 (53.4–1299)^b^	20.182	**< 0.001**
Feed (µg/L)	642.55 ((− 93.2)–4620)	260.45 (1820–960.1)	155.55 ((− 65)–389.8)	6.006	0.050

H = Kruskal–Wallis H Test, *p* < 0.05 = statistically significant

The letters ^a,b^represent the post-hoc result. Differences between different letters are significant

Table 4 shows compares boron concentrations (serum, urine, drinking water, and feed) across different distances (0–5 km, 5–15 km, and 15–30 km). Serum boron concentration is highest at 0–5 km (0.54 µg/L), lower at 5–15 km (0.37 µg/L), and moderate at 15–30 km (0.45 µg/L), with a statistically significant difference (H = 9.929; *p* = 0.007). Although urine boron concentrations vary across distances (0–5 km: 10452.5 µg/L; 5–15 km: 3319.5 µg/L; 15–30 km: 4929 µg/L), the differences are not statistically significant (H = 5.373; *p* = 0.068). Drinking water boron concentration is significantly high at 0–5 km (1285 µg/L), while it decreases substantially at 5–15 km (93.20 µg/L) and 15–30 km (73.75 µg/L) (H = 20.182; *p* < 0.001). In feed samples, boron concentration is markedly higher at 0–5 km (642.55 µg/L) and decreases at greater distances (H = 6.006; *p* = 0.050).

**Fig. 2:**
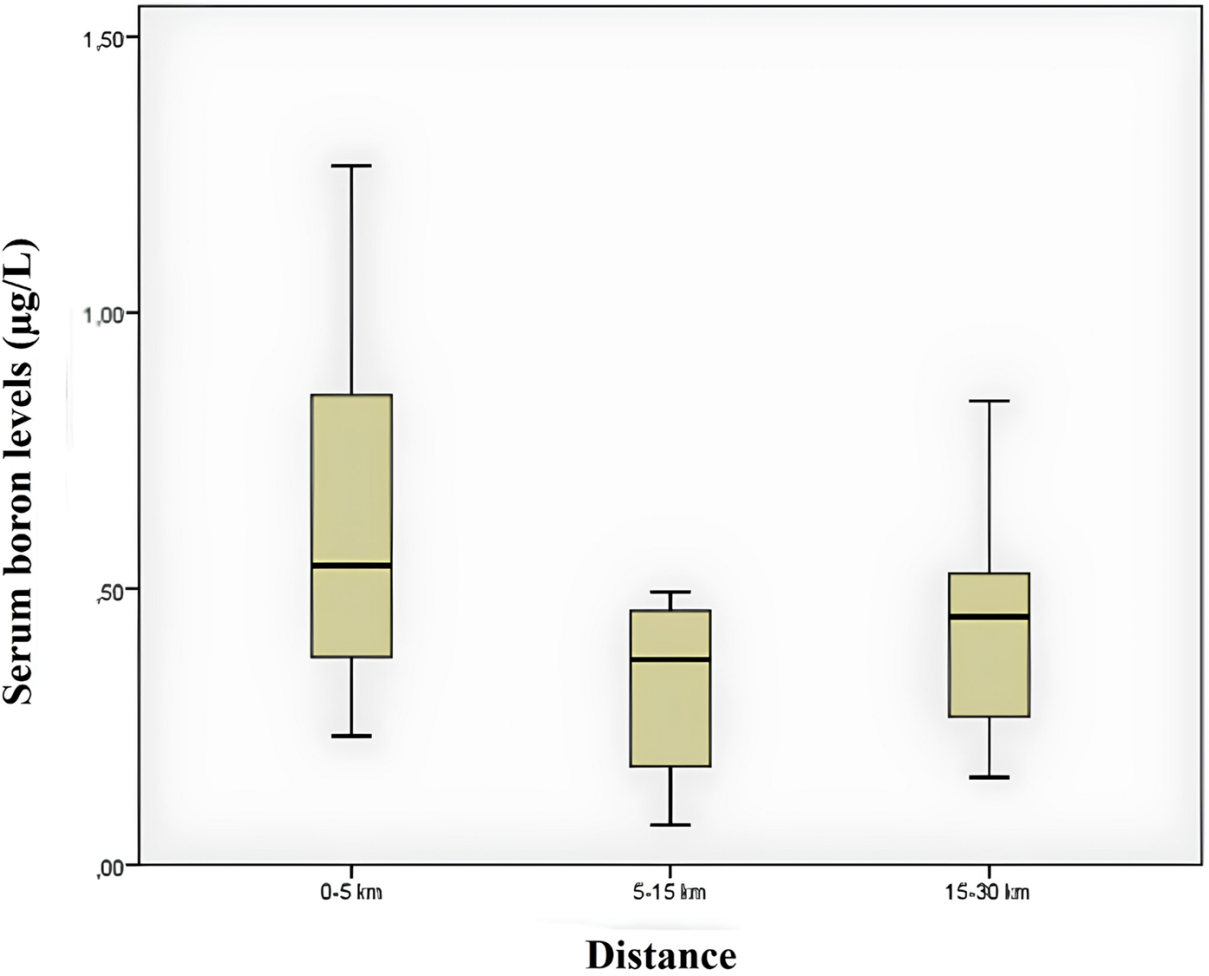
Box plot of serum boron measurements by distance

Figure 2 shows a box plot comparing serum boron measurements by distance. When the graph is examined, the median serum boron value is higher in samples taken from 0 to 5 km distance. Looking at the interquartile range (IQR), it is seen that it narrows with distance. This indicates that the variability in serum boron levels decreases with increasing distance.

**Fig. 3:**
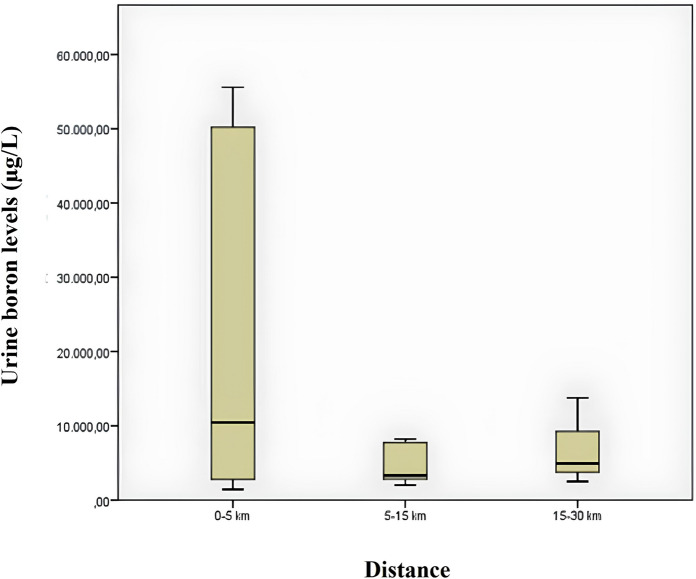
Box plot of urinary boron measurements by distance

Figure 3 shows a box plot comparing urinary boron measurements by distance. When the graph is examined, the median urinary boron value is higher in samples taken from 0 to 5 km distance. Looking at the quartile span, it is seen that it narrows significantly with distance. This indicates that the variability in urinary boron levels decreases with increasing distance. Despite the large visual difference in the graph, Kruskal–Wallis test results show that there is no statistically significant difference between the distances. There could be several different reasons for this. One of them is that box plots show medians, quartiles and outliers but not the entire distribution. Therefore, especially if the within-group variability is high (quartile spacing is large), the median values may be different but the overall distribution may overlap to a large extent. Another reason is that outliers can greatly affect the results of the analysis, especially in small samples (µg/L).

**Fig. 4:**
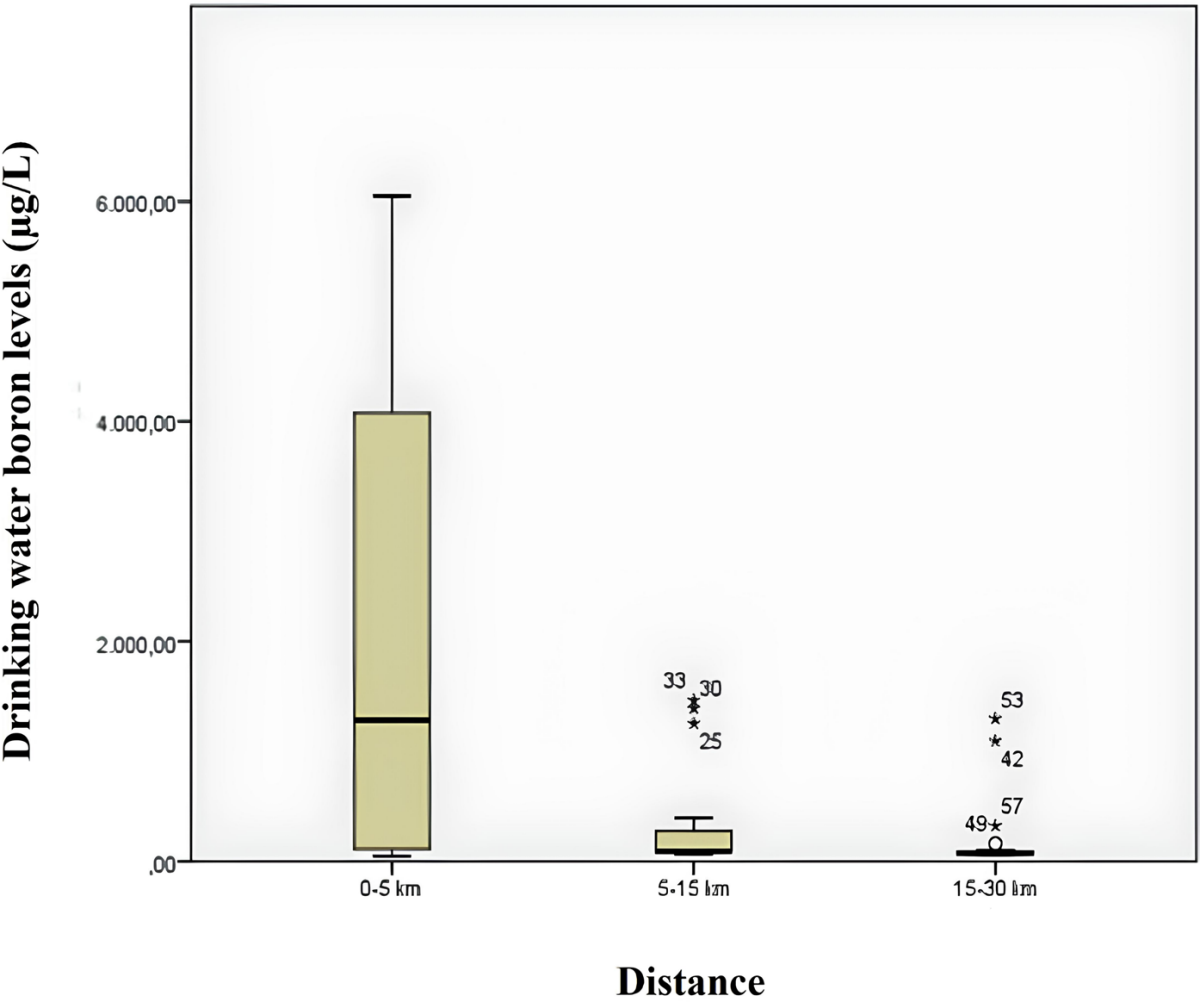
Box plot of drinking water boron measurements by distance

Figure 4 shows a box plot comparing boron measurements of water samples according to distance. When the graph is examined, it is seen that the median urinary boron value is higher in samples taken from 0 to 5 km distance. Looking at the quartile span, it is seen that it narrows significantly with distance. This shows that the variability in water boron levels decreases with increasing distance. Looking at the graphs for the distances of 5–15 km and 15–30 km, it is possible to infer that the variability in boron levels at these distances is extremely low, that is, the measurements at these distances are close to each other. On the other hand, there are some outliers.

**Fig. 5:**
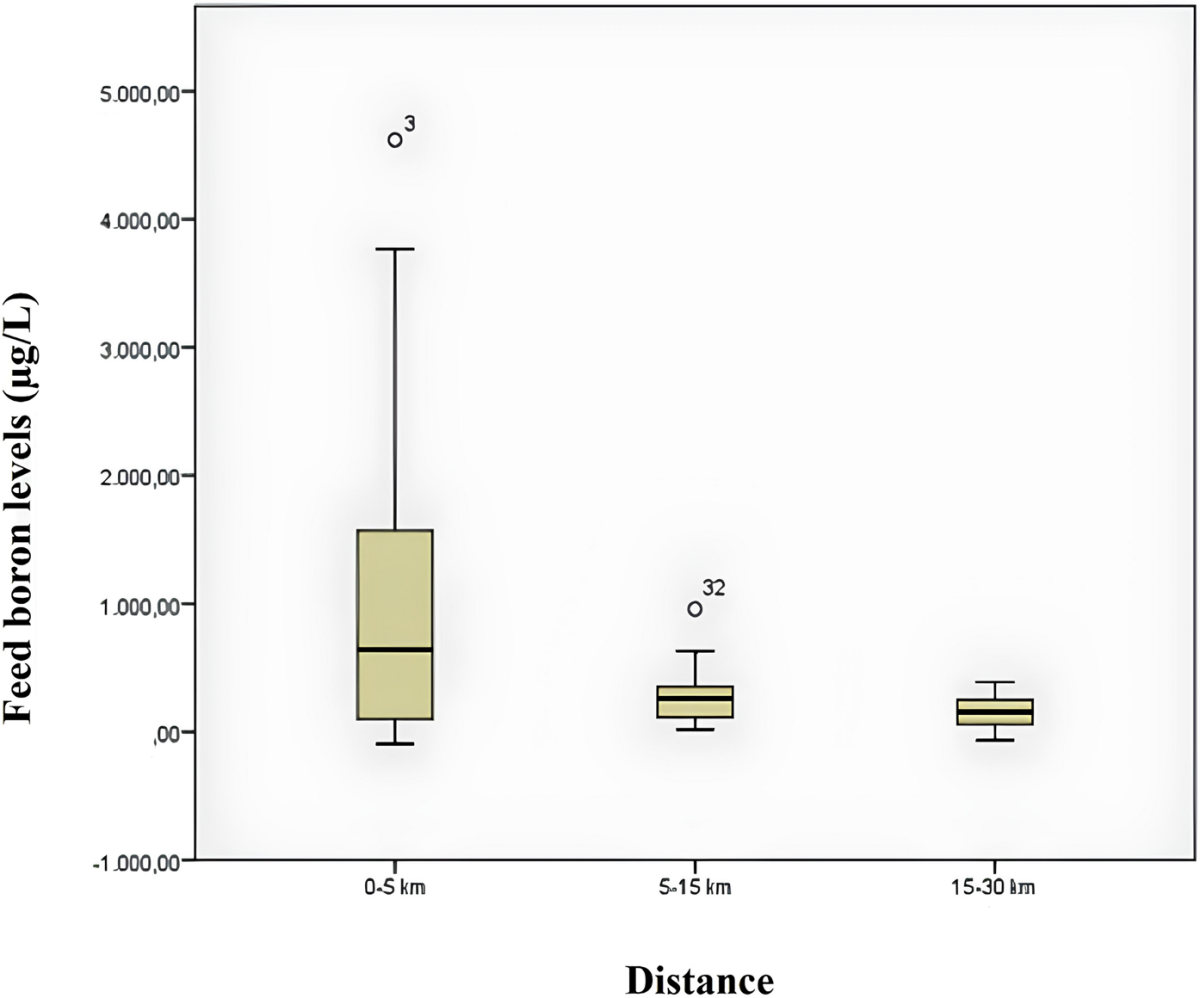
Box plot of feed boron measurements by distance

Figure 5 shows a box plot comparing boron measurements of feed samples according to distance. When the graph is examined, it is seen that the median urinary boron value is higher in samples taken from 0 to 5 km distance. Looking at the quartile span, it is seen that it narrows significantly with distance. This shows that the variability in boron levels in feeds decreases with increasing distance. When the graphs for the distances of 5–15 km and 15–30 km are examined, it is seen that the quartile spacing is quite narrow.

## Discussion

There are various limitations in studies on boron exposure. The most important of these limitations are geographical conditions and dietary habits. Boron is not included in USDA nutrient databases and there is no comprehensive analytical database on the boron content of specific foods (meat, milk). However, meat and dairy products have been designated as boron-poor foods. Despite this, they contribute significantly to the daily dietary intake of boron, as they are more common in the diet (Kuru et al., 2019). Therefore, boron exposure of animals, which are the source of these foods, is very important. Consequently, the reference dose or recommended dietary boron intake has not yet been clearly established. Water sources and boron concentration in food are the two main sources of boron intake. Data on the boron content of drinking water available for human and animal consumption in Turkey are also lacking. Therefore, determination of boron levels in drinking water will provide useful baseline information for the assessment of boron intake through drinking water and for further studies to protect and improve public health. Numbers of scientific studies on biological effects of boron compounds show a steady increase. This includes studies on human safety aspects, which are triggered by regulatory issues (Bolt et al., 2017; Khaliq et al., 2018). This is the first study to investigate boron levels at different distances from the mine site and the most comprehensive study in terms of the numbers of the samples collected.

### Water samples

Reliable and good quality drinking water is also essential for human health and animal production. Water sources account for the largest share of environmental contamination. In recent years, due to the emergence of global industrial demand for boron and the further spread of boron in water supply, quality criteria have become more stringent and have begun to progress rapidly. In 2020, a study investigated boron concentration in 335 tap water samples collected from 75 cities in Turkey. The mean boron level in the cities of Turkey was found to be 0.2 mg/L. This is expressed as lower than the limit value given by WHO (2.4 mg/L) and the EU (1 mg/L) (Kuru et al., 2019). In a study conducted in Kütahya, one of the boron mining areas, 382 water samples were collected from different sources. The average boron amount in all water samples was 10.204.08 mg B/l. This value is much higher than the limits determined as 0.3 mg B/l by WHO and US Environmental Protection Agency (EPA) (Çöl & Çöl, 2003). In one study, water samples were taken from locations identified as study areas. The findings were divided into 3 categories: (a) those with water sources containing boron concentrations up to 2.0 mg l-1; (b) those with boron concentrations up to 10mgl-1; and (c) those with higher boron concentrations. These waters are reportedly not used for irrigation. Iskele and its surroundings (our 0–5 km group), where the boron mine site is located, was the group where the highest boron concentrations were measured (Simsek et al., 2003).

In a study investigating the relationship between boron exposure and fertility, values between 2.05 and 29 mg B/L were considered high boron zones. This is very dangerous, especially for human health. In this study, fertility figures did not differ from other regions. However, it is important that these studies are repeated with today’s possibilities and that the results are up-to-date (Saylı et al., 1998). In another study, drinking water samples were collected both near from the boron-rich area (Bigadic county in Balikesir city in Turkey) and far from this area (control area: city centre of Balikesir). The boron levels of samples, by ICP-MS analysis, in boron-rich area have been found to be between 1.42 and 6.51 mg/L, while it has been found between 0.008 and 0.011 mg/L in control area (Korkmaz et al., 2007).

For this study, average boron levels were determined as 2134.68 µg/L for SD, 327.05 µg/L for MD and 200.75 µg/L for LD. These values are consistent with other studies conducted at the mine sites. A valuable result is that the boron concentration in water increases as it approaches the mine site. In addition to other studies, the comparison of the effect of 3 different distances on boron concentrations emphasizes the importance of the distance to the mine in environmental pollution. In this respect, this study addresses a topic that has not been investigated before.

### Feed samples

Boron is a micronutrient with a limited range of deficiency and toxicity in plants. Boron transporters in plants are involved not only in boron uptake but also in its removal in case of boron toxicity (Khan et al., 2022; Landi et al., 2019). Boron is required for almost all plant functions, including quality maintenance, production, development and optimum growth (Shireen et al., 2018). Studies investigating boron concentrations in plants are generally lacking. At studies information presented has some limitations. The most important of these is the lack of data on the boron content of soils, even though the study areas are part of the same geological region, i.e. a boron-rich region. Some fluctuations can therefore be expected between regions, reflecting boron concentrations in the plants growing there. Daily boron intake depends on nutritional tradition and food consumption frequency. In Turkey, there are limited number of studies on the boron content of foods offered for human and animal consumption.

In a study, samples were taken from fields in some boron-intensive regions (Bigadiç, Susurluk, Mustafa Kemal Paşa, Kırka, Emet, Hisarcık) and compared with the control region (Ankara). Although high boron concentrations were detected in some samples, it was reported that there may be many reasons for this and that it does not pose a health risk. In this study, it was reported that since various factors play a role in boron uptake in plants, high levels should be considered normal (Simsek et al., 2003).

A study was conducted to investigate boron concentrations in 42 different foods including various food types (vegetable, animal products vb.). Boron concentration in foods ranged between 0.06 and 37.2 mg/kg. Especially high boron concentrations have been reported in cow’s milk, olives and green beans. The reason for these differences may be due to the origin of the foods and different methods for boron determination. Among the sources is Balıkesir where borate is produced (Kuru et al., 2019). There are no studies on foods intended for direct animal consumption. Therefore, no comparison could be made. Our study is the first of its kind in the literature.

In this study, the mean boron levels were determined as 1151.43 for SD, 293.71 for MD and 159.46 for LD. According to these results, it was determined that the distance to the mine site affected the boron concentration. Boron was detected in silages offered for animal consumption. This situation is of great importance for environmental contamination and animal health. It was also considered as a step of exposure.

### Blood samples

Boron compounds are rapidly converted to boric acid in the gastrointestinal tract when ingested orally. Almost all of it is absorbed and distributed to tissues via blood (Kuru & Yarat, 2017). There are very few studies on the distribution of boron in the body. Levels of boron were measured in the organs and blood of individuals who perished in an accident. The concentrations were found to be 0.5 µg/g and 1 µg/g in the kidneys and liver, respectively. Furthermore, boron concentrations of 30 ng/ml were reported in synovial fluid and 28 ng/ml in blood (Sutherland et al., 1999).

In one study where, men and women residing in the İskele and Osmanca villages of Bigadiç participated. B concentrations in some drinking water sources in Iskele were much higher (up to 18.04 mg B/L) than the limits set by both EU (1 mg B/L) and WHO (2.4 mg B/L) drinking water guidelines, indicating significant environmental exposure. Blood B concentrations of study participants were found to be (< 100 mg B/g blood), moderate (100–150 ng B/g blood), high (> 150–450 ng B/g blood) and very high (> 450 ng B/g blood). The study also measured daily boron exposure levels. According to the results; boron concentrations in blood samples were significantly correlated with the daily exposure levels. (Duydu et al., 2018).

Whole blood and urine boron concentrations were measured in pregnant women exposed to boron through drinking water. Boron ranging between 377 and 10,929 µg/L in drinking water showed a strong correlation with whole blood and urine in this study. In the study, each 100 µg/L increase in serum boron corresponded to newborns that were 0.9 cm shorter and 120 g lighter (Igra et al., 2016).

In a recent epidemiological study, boron exposure in mining areas and processing plants in China and Turkey was measured to be as high as 41.2 and 47.17 mg/day, respectively. This level is well above the recommended dose for developmental and reproductive effects of boron (9.6 mg B/day–20.3 mg B/day, respectively). In the same study, the blood boron concentration of boron-exposed male workers was 570.6 ng B/g (ppb). As a result of the study, it was reported that the effects of boron in daily life may be shaped in a concentration-dependent manner and clear data have not yet been presented (Duydu et al., 2023).

In this study, the mean boron levels were determined as 0.63 µg/L for SD, 0.32 µg/L for MD and 0.41 µg/L for LD. Comparisons could not be made as there was no sample research. However, since the SD areas are common, it is considered to be compatible with the present study. There is no study in the literature examining the change in boron volume with respect to the distance to the mine site.

### Urine samples

Boron in the body in the form of boric acid is the only boron compound detected in urine. Besides urine, excretion can also occur through saliva, feces, sweat and respiration. However, boron in urine is considered as an indicator of oral dose (EFSA, 2004; Moseman, 1994). The concentration in the urine of animals exposed to boron orally from water and food, as well as inhaled from the air, is of interest. There are no studies evaluating the effects of location relative to the mine site in this area. In this study, the mean boron levels were determined as 22685.00 µg/L for SD, 4998.35 µg/L for MD and 6265.20 µg/L for LD.

155 people working in boron processing plants in Bandırma are divided into 3 groups as low (7.39 ± 3.97 mg/day), medium (11.02 ± 4.61 mg/day) and high (14.45 ± 6.57 mg/day) boron exposure with air, water and food. The group with low daily boron exposure had the least amount of urine boron (5.01 ± 2.07 ppm) and serum (72.94 ± 15.43 ppb) boron for the least period of time, while the group with high boron intake had the highest amount of urine boron (9.83 ± 5.13 ppm) and serum (223.89 ± 69.49 ppb). Moreover, a positive and significant difference was identified between ambient and serum boron levels (Duydu et al., 2011).

## Conclusion

Industrialisation, uneven urbanisation and the proximity of mining areas to urban centres have all played a major role in the deterioration of the ecological balance. High levels of pollutants in water, soil, air and, indirectly, food, cause pollution and pose a risk to all living creatures, including humans and animals. All substances in high concentrations pose a risk in terms of toxicity and are passed through the food chain to plants and animals and finally to humans, the last link in the chain.

Turkey has the highest share of boron reserves with 73.5%, the existence of which has been known for 6000 years. The first boron deposit in Turkey was found in Balıkesir province in 1815. While some of boron and its compounds are used directly as minerals, some of them are used in many different fields such as industry, agriculture, glass industry and health. However, boron is an element with a very thin boundary between deficiency and excess. For this reason, the effects of intense exposure in the regions where it is mined and processed are important and curious. In addition, it enters our lives indirectly through consumption of contaminated of animal products and plants, and directly through many substances offered for human consumption.

The province of Balıkesir, which is a boron mining region, was chosen for our research. Urine, blood, water and feed samples from animals living at different distances from the mine were analysed. The results of the analyses show that the amount of boron decreases as the distance from the mine decreases. This situation suggests that boron may with pose health problem to for plants growing in the field and for animals and humans living there. It is therefore worth investigating the effects of boron on the environment and living organisms. However, there are some limitations to our study. One of the limitations of this study is that only one animal species in the region was analysed. This study, which was only carried out on cattle, could not determine the boron exposure of other animals (sheep, cats, dogs, etc.) and humans in the region. It is recommended that further extensive studies be conducted on other farm animals, poultry, or aquatic organisms. The present study investigated water and feed boron exposure. However, a fundamental limitation of the study was the absence of data on the boron levels of products derived from the animals included in the study. This included milk, meat and offal, which are of particular relevance to public health in view of their frequent consumption. Animal manure is also used in the region. Faecal samples from the cattle included in the study were not analysed for boron. All these situations remain uncertain in terms of public health. A more comprehensive study should be carried out, including boron concentrations in foods such as meat, milk, etc., which can be transferred from boron-exposed animals to humans. This comprehensive approach enables a holistic analysis of human and environmental exposure. Another limitation of the study is that boron levels in the air were not determined because we did not have the necessary equipment to measure air levels. Airborne exposure levels may be subject to variation according to seasonal conditions; consequently, measurements can be taken at specified intervals in order to obtain information regarding periods of heightened risk.

## Data Availability

No datasets were generated or analysed during the current study.
